# AFP-producing hepatoid adenocarcinoma of the stomach: a case report

**DOI:** 10.1186/1757-1626-2-9296

**Published:** 2009-12-09

**Authors:** Panagiotis J Vlachostergios, Ioannis A Voutsadakis, Sotirios Barbanis, Foteini Karasavvidou, Christos N Papandreou

**Affiliations:** 1 Division of Medical Oncology, University Hospital of Larissa, University of Thessaly, School of Medicine, Larissa, Greece; 2 Department of Pathology, University Hospital of Larissa, University of Thessaly, School of Medicine, Larissa, Greece

## Abstract

Hepatoid gastric adenocarcinoma is a distinct variant of gastric carcinoma which represents a comparatively small percentage of the disease and in many cases is producing high serum alpha-fetoprotein (AFP). We report a case of an 85 year old woman who presented with epigastric and right upper quadrant pain and was found in a CT scan to have multiple liver nodules and a gastric antrum mass as well as an elevated AFP level of 155000 IU/ml. An endoscopic biopsy of the antral mass showed hepatoid variant of gastric adenocarcinoma. The patient refused any further treatment and died 4 months after diagnosis. Hepatoid gastric adenocarcinoma is considered to have a poor prognosis, although cases with survival of several years have been reported. Poor outcome in most of the cases is due to the fact that, as in our patient, metastatic disease is already present at diagnosis.

## Background

Hepatoid gastric adenocarcinoma is comparatively rare and most case reports come from the Far East [1-4] while reports from Europe and North America are fewer [1,5-9]. Although some authors estimate hepatoid type adenocarcinomas of the stomach to represent 1.3 to 15% of gastric carcinomas, these percentages are, at least for western countries, an overestimation and only a few hepatoid gastric adenocarcinomas have been reported in the recent literature. AFP overproduction by these carcinomas happens in most but not all the cases [1] and may help direct the diagnosis in cases that it is elevated. In this case report, an 85 year old woman with an AFP-producing hepatoid type gastric adenocarcinoma is described.

## Case presentation

**Figure 1:**
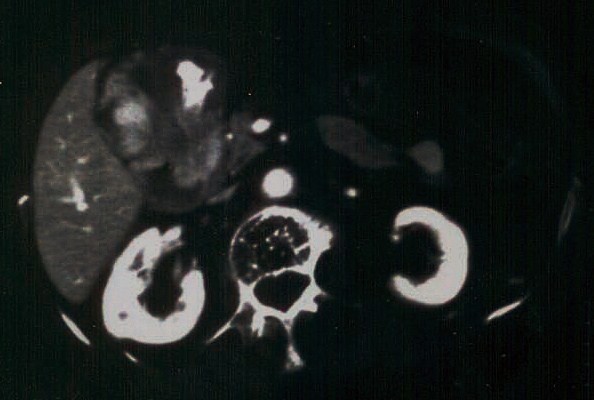
**Abdominal CT scan showing an antral tumor and liver metastases**.

**Figure 2:**
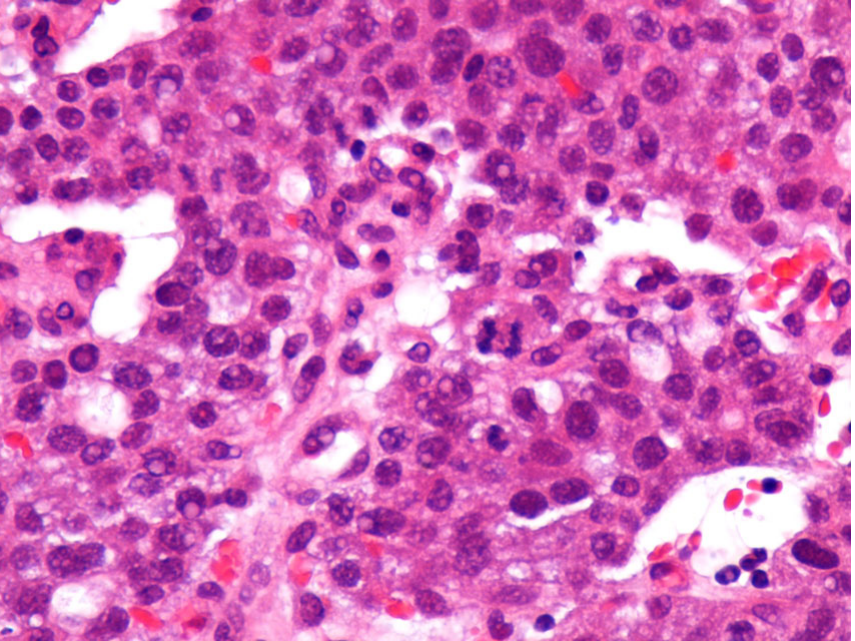
**Histopathology**. Poorly differentiated gastric adenocarcinoma with hepatoid features, composed mainly of trabecular and focal glandular formations. HE stain × 400.

**Figure 3:**
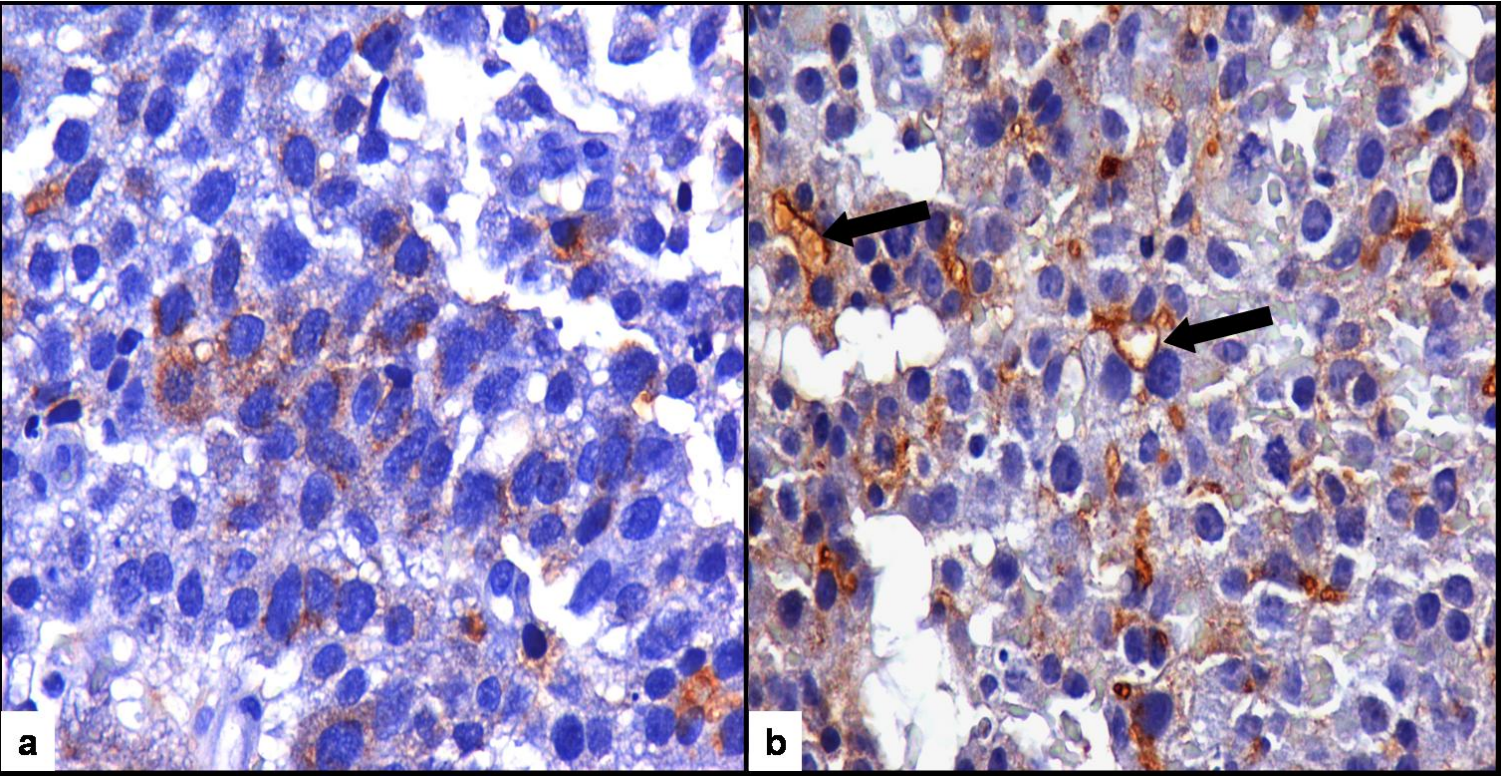
**Histopathology**. **a**. Immunohistochemical cytoplasmic positivity for a1-fetoprotein, × 400. **b**. Immunohistochemical "canalicular" pattern of staining for polyclonal carcinoembryonic antigen (CEA), × 400 (arrows).

An 85 year-old woman presented with epigastric and right upper quadrant abdominal pain of 3 days duration. The patient had no relevant medical history, except for mild hypertension and an excised squamous scin carcinoma of the right hand 4 years previously. Review of systems disclosed weight loss of 5 kgs in 2 months but no hematemesis, hematochezia or melena. Physical examination was positive for mildly anaemic sclerae. Laboratory investigation showed a hematocrit of 34% with hemoglobin of 11.5 g/dl, MCV of 113 fl and RDW of 13.5%. The AFP level was very elevated at 155000 IU/ml. Other tumor markers were normal. Hepatitis B and C panel was negative. An esophagogastroduodenoscopy revealed a gastric antrum mass, and an abdominal CT showed the mass to measure 7 cm and disclosed several liver metastases (Figure 1). Histopathologic examination of the antral mass biopsy showed poorly differentiated adenocarcinoma with hepatoid features. The carcinoma was composed of polygonal tumor cells with abundant eosinophilic cytoplasm and round nuclei occasionally with obvious nucleoli and high mitotic activity. The tumor cells were arranged mainly in a trabecular pattern while a smaller area of glandular formations was also seen (Figure 2). Some cells showed cytoplasmic mucin positive for PAS-diastase (Periodic acid-Schiff-diastase) histochemical stain. Immunohistochemically, the neoplastic cells revealed diffuse positivity for a mixture of cytokeratins 8, 18 and 19. Many neoplastic cells interestingly showed cytoplasmic positivity for a1-fetoprotein (Figure 3a). Additionally, a "canalicular" pattern of staining was seen with polyclonal carcinoembryonic antigen (CEA), which is a characteristic hepatoid feature (Figure 3b). The tumor cells were negative for the hepatocyte specific marker hepatocyte paraffin-1 (Hep Par-1). These immunohistochemical findings combined with the morphological features described above, supported a diagnosis of hepatoid variant of gastric adenocarcinoma. The patient and family refused any treatment and returned home on oral analgetics as needed. She died of her disease 4 months after admission.

## Discussion

Gastric hepatoid adenocarcinoma (GHAC) with elevated serum AFP, first described by Ishikura et al. 1985 [10], is a rare gastric cancer subtype seen more frequently in older patients, aged 60-70 years, with an elevation of serum AFP level. The antrum of the stomach is the most common site [1]. There are no specific symptoms and signs but generally, epigastric pain and fatigue are observed most frequently [1]. Liver and lymph node metastases are frequently found at diagnosis [11]. The primary tumor usually appears as a large, necrotic and moderately vascular mass in CT scan [12]. Histopathologically, the tumor is composed of two closely related areas, hepatoid-like foci and adenocarcinomatous. Tumor cells in hepatoid foci resemble the morphology of hepatocellular carcinoma (HCC), and immunohistochemically can be positive for AFP, alpha-1 antitrypsin (AAT), alpha-1 antichymotrypsin (ACT) and albumin (ALB). Additionally, polyclonal CEA shows a "canalicular" pattern of staining which is a characteristic hepatoid feature. The adenocarcinomatous component may be well or poorly differentiated, often with clear cells and papillary pattern [13,14].

Recently, glypican 3 (GPC3) has been evaluated as a sensitive marker for AFP-producing gastric carcinoma (GC) and its hepatoid component, and the authors support its usefulness for identifying this aggressive subgroup of GC [15]. Both the primary tumor and the metastatic liver lesions of GHAC, when present, need to be differentiated primarily from HCC. Generally, in HCC, neighboring cirrhotic lesions can be seen and tumor cells are positive for Hep Par-1, a sensitive and specific immunohistochemical marker for hepatocyte differentiation, whereas in metastatic GHAC Hep Par-1 is often negative and neighboring cirrhotic lesions are seldom seen [14]. P53 protein immunohistochemical detection in GHAC has also been demonstrated. In classic adenocarcinoma of the stomach, p53 protein is frequently expressed and often correlates with a poor prognosis. In contrast, overexpression of p53 is a rare event in HCC [16]. A most recent finding indicates the use of the palate, lung, and nasal epithelium carcinoma-associated protein (PLUNC) immunostaining as a novel marker that distinguishes GHAC from primary HCC [17]. However, the presence of markers of hepatoid differentiation cannot be used as the sole diagnostic criteria for GHAC but must always be accompanied by analogous morphological patterns [18]. In differential diagnosis, other AFP-producing gastric tumors as well as a metastasizing germ cell tumor should also be excluded [9].

In terms of treatment, there are only sparse data in the literature pertaining specifically to GHAC and the disease should be in general treated similarly to common gastric adenocarcinoma. Surgical management of an early primary tumor, if feasible, is the indicated approach. Adjuvant chemotherapy and radiotherapy should be given according to current gastric cancer indications, despite the fact that no specific data on adjuvant treatment of GHAC are available. Palliative chemotherapy is the mainstay of treatment in inoperable, recurrent or metastatic cases. Concerning specific chemotherapy regimens, there are again few data specific for this sub-type of cancer [1]. A case report of an AFP-producing hepatoid gastric cancer associated with multiple liver metastases that was successfully treated with paclitaxel-based chemotherapy has recently been published [19].

Liver metastasectomy of completely resectable metastases may be considered in selected cases. The first case of a long-term (11 years) survival of AFP-producing gastric cancer with successfully resected metachronous liver metastasis and gastric remnant carcinoma has recently been reported [20]. In such cases, long-term follow-up and close observation are required to find any symptoms of recurrence after gastrectomy, and early CT or MRI of clinically suspect areas should be performed.

## Conclusion

A multidisciplinary treatment approach modelled from other types of cancer should be instituted to obtain optimal results in hepatoid adenocarcinoma of the stomach. Further investigation in the molecular pathogenesis of hepatoid gastric adenocarcinoma is also of importance in order to elucidate its origin and introduce novel treatments.

## Abbreviations

CT: computed tomography; MCV: mean corpuscular volume; RDW: red blood cell distribution width; MRI: magnetic resonance imaging.

## Consent

Written informed consent was obtained from the patient for publication of this case report and accompanying images. A copy of the written consent is available for review by the Editor-in-Chief of this journal.

## Competing interests

The authors declare that they have no competing interests.

## Authors' contributions

PJV and IAV contributed equally to this work. PJV performed the research and wrote the paper. IAV conceptualized and co-authored the paper, provided clinical care and performed research. SB and FK performed research and provided histopathological data. CNP revised the paper. All authors read and approved the final manuscript.
